# Oral acute and sub-acute toxic effects of hydroalcoholic *Terminalia chebula* Retz and *Achillea wilhelmsii* extracts in BALB/c mice

**DOI:** 10.1051/bmdcn/2019090425

**Published:** 2019-11-21

**Authors:** Mahnaz Jafari, Kourosh Manochehri Naeini, Zahra Lorigooini, Rasool Namjoo

**Affiliations:** 1 Medical Plants Research Center, Basic Health Sciences Institute, Shahrekord University of Medical Sciences Shahrekord Iran; 2 Department of Pathology, School of Veterinary Medicine, Islamic Azad University Shahrekord Iran

**Keywords:** *Achillea wilhelmsii*, Acute toxicity, Animal Model, Sub-acute toxicity, *Terminalia chebula*

## Abstract

Background: This study examined the acute and sub-acute toxic effects of *Terminalia chebula* and *Achillea wilhelmsii* extracts on the murine model.

Methods: In both phases, mice were assigned to intervention and control groups. At the end of study, the liver, kidney, and heart tissues were collected for histopathological studies.

Results: In the acute phase of the study, the safe dose was ≤5000 mg/kg for both extracts. In sub-acute phase, LD50 (95% CI) of *Achillea wilhelmsii* extract was determined ≥5000 mg/kg and that of *Terminalia chebula* extract 2754.436 (2438-3114) mg/kg. The highest dose of *T. chebula* extract induced few histopathological changes.

Conclusion: It will be useful to gain information on the minimum lethal doses of *T. chebula* and *A. wilhelmsii* to adopt safe doses of the two plants.

## 1. Introduction

Nowadays, due to the increasing use of medicinal herbs to treat various diseases, the study of the therapeutic and negative effects of these drugs has attracted the attention of researchers. However, most pharmaceutical studies have mainly focused on the beneficial properties of the plants and their safe and toxic doses and side effects of them have not been sufficiently investigated. So to ensure the safety of herbal drugs, the study of the long-term and short-term toxic effects of these drugs is essential [1, 2]. Acute or lethal toxicity refers to a chemical compound’s ability to lead to death relatively soon after being orally ingested, or after being exposed, e.g., as a gas in the air, for four hours. The period has conventionally been expressed in minutes, hours, and weeks (up to two weeks), and has rarely been defined as longer than the mentioned periods. Sometimes the LD50 (median lethal dose) is referred to as the mean lethal dose. The LD50 of a certain compound is the dose of that compound that kills 50% of the members of a tested population (e.g. laboratory rats or mice) after specified test duration through the already described methods. The purpose of acute toxicity study is to obtain information on biological and chemical activity and mechanisms of action, which is the basis of toxicology and chemical classification. In most studies that have been conducted on toxicity rate of medicinal agents in laboratory animals, test agents are inoculated orally. Studies in this area are important with respect to drugs, foods, and accidental poisoning [3, 4]. LD50 values can be very small or vice versa, comparable to other toxicity scales. Toxicity measurement methods are numerous, most important of which are Gosselin scale and Hodge-Sterner scale [5]. As no study has yet been conducted on the sub-acute and acute toxicity of *Terminalia chebula* Retz and *Achillea wilhelmsii*, the study was aimed to investigate these aspects of the extracts. *T. chebula* is from the Combretaceae family and is a herbaceous, perennial, and juicy plant natively occurring in India and Southeast Asia. Due to the presence of secondary metabolites, this plant has been considered a herbal drug. The fresh and dry fruit of this plant has a lot of phenolic compounds and strong antioxidant properties [6, 7]. The antibacterial, antifungal, antimutagenic, immunomodulatory activities, antiviral, antioxidant, anti-cancer, antimalarial, and anti-diabetic properties of *T. chebula* were demonstrated in different studies [8–18]. Yarrow (*Achillea wilhelmsii*), belonging to the Asteraceae family, has been applied in Iranian and other traditional medicine to treat some diseases. The species of the Achillea genus have a considerable amount of essential oil. *A. wilhelmsii* has secondary metabolites including terpenes, flavonoids, alkaloids, tannin, and lignin [19, 20]. Based on studies, the anti-hypertensive, anti-inflammatory, antibacterial, anticancer, and antioxidant effects of *A. wilhelmsii* have been proven. In addition, this plant can lower cholesterol and glucose levels [21–26].

## 2. Materials and methods

### 2.1. Collecting plant samples and extraction

The plants were purchased from medicinal plant stores and identified as the plants of interest by botanists, and two voucher specimens (no. 27 and 304) were deposited for *T. chebula* and *A. wilhelmsii*, respectively, at the Herbarium of Shahrekord University of Medical Sciences Chaharmahal va Bakhtiari Province. In order to prepare extracts, maceration was done in 70% ethanol during 72 hours and the mixture was refined using Buchner funnel and Whatman number 1 paper. The resulting extract was concentrated in vacuum in a Rotary evaporator at 35°C. The extract was then incubated at 40°C to dry and ultimately, the dried extract was left under -20°C temperature till it was used [27].

### 2.2. Determination of Total Phenolic Content

The total phenolic content was measured by the Folin-Ciocalteu colorimetric method. First, Gallic acid at different concentrations was prepared and the standard curve was plotted by reading their absorbance at 765 nm. Then, 100 μL of plant sample stocks was combined with 500 μL Folin-Ciocalteu and 1000 μL distilled water. The resulting mixture was well stirred and kept under room temperature for one minute. Afterwards, 1500 μL 20% sodium carbonate was introduced and the resulting mixture left at room temperature for 2 hours. The optical absorbance of the samples was recorded at 765 nm spectrophotometrically and the data were reported in mg gallic acid equivalent (GAE)/g dry weight of the extracts [28].

### 2.3. Measuring total flavonoid content

The total flavonoid content was measured spectrophotometrically based on flavonoid-aluminium complexation, described by Lamaison and Carnat. Briefly, 1 mL extract solutions were combined with 1 mL 2% ethanolic AlCl3 and 3 mL 5% potassium acetate. The mixture was then left at room temperature for 15 minutes, and finally, the optical absorbance of the mixture was spectrophotometrically read at 430 nm, and the results were presented as mg rutin equivalent/g dry weight of the plant extracts [28].

### 2.4. Measuring antioxidant activity

The antioxidant activity was measured by the protocol of Kirby and Schmidt in 1996. The main point of this protocol is discolouration of 2, 2-diphenyl-1-picrylhydrazyl (DPPH) and reduction of absorbance at 517 nm due to the influence of the antioxidant compound. The inhibition of absorbance at 517 nm was plotted to obtain antioxidant concentration. In this test, butylated hydroxytoluene (BHT) was used as standard [14, 29].

### 2.5. Animals

In this study, the animals that used were male BALB/c mice (25-30 g, aged 7-8 weeks), which were kept at 12/12-h light/dark cycle under (22 ± 2)°C. The animals were transferred to the experiment environment one week before the tests. This study protocol was confirmed by Shahrekord University of Medical Sciences Ethics Committee (ethics code: IR.SKUMS.REC1394.196)

### 2.6. Acute toxicity test

To conduct this step of research, the method described by lorke et al was used [30]. This step of the research was performed in two phases: First, the mice were assigned to 10 groups of 5 each randomly. For each of the extracts, one group was considered as controls and four groups as test groups. Then, in the control group, 0.5 mL of sterile normal saline was administered via oral gavage and the mice in different case groups were separately treated with 10, 156.25, 312.5, and 625 mg/kg of the extracts of *A. wilhelmsii* and *T. chebula*. The extract doses were selected according to the OECD Guidelines for the Testing of the Chemicals. At this stage, all administrations were performed as a single oral dose. Then, the the signs of toxicity were examined in mice (behaviour, respiratory pattern, cardiovascular symptoms, motor activity, reflex, and fur and skin changes) and mortality at 1, 2, 4, 8, 12, 24, 48, and 72 h intervals. Because there was no mortality at the intervals, the second stage of the experiment started. In this stage, the mice were assigned to 8 groups of 5 each (two control groups and three case groups for each extract). Then, in the control group, 0.5 ml of sterile normal saline was administered by the mentioned method and the mice of the three case groups were separately given 1250, 2500, and 5000 mg/kg of each of extracts and the mice were examined for toxicity signs and mortality at the above-mentioned intervals [3].

### 2.7. Subacute toxicity test

Mice were randomly assigned to 14 groups of 5 each (one group considered to be control and 6 groups to be cases treated with each studied extract separately). Then, the mice in the control group orally received 0.5 ml of sterile normal saline for 14 days, and the mice in case groups were separately treated with 156.25, 312.5, 625, 1250, 2500, and 5000 mg/kg of extracts of *A. wilhelmsii* and *T. chebula*. The mice were monitored for toxicity signs and mortality on a daily basis for 30 days [31–33].

### 2.8. Histopathological investigation

At the completion of the study, the mice were anaesthetized with chloroform and pieces of liver, kidney, and heart were removed so that they could be histopathologically examined. After the tissues were placed in 10% formalin solution and paraffin blocks were prepared, 5 μm sections were prepared from the tissues. To conduct histopathological investigations, hematoxylineosin staining was conducted on the sections and the lams were microscopically examined for possible lesions. To save time, the aforementioned measures were performed in control mice and mice receiving doses of 2500 and 5000 mg/kg of the extracts. If there were tissue changes in this stage, histopathological examinations were also performed in mice receiving lower doses of extracts [34].

### 2.9. Statistical analysis

Data analysis was conducted by the SPSS version 20 by Probit regression and P less than 0.05 was assumed significance level.

## 3. Results

### 3.1. Total phenolic content, flavonoid content and antioxidant activity of *T. chebula* and *A. wilhelmsii*

**Fig. 1:**
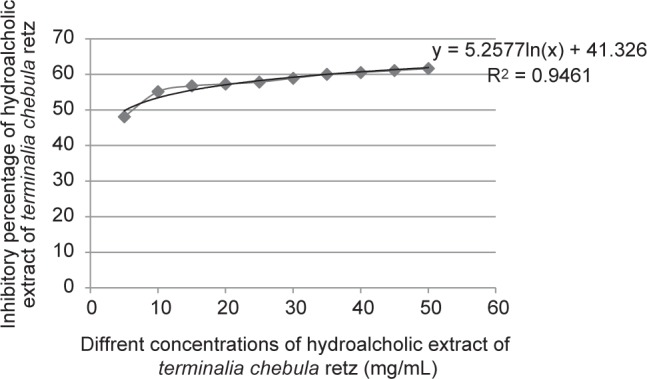
Percentage of DPPH free radicals inhibition by *Terminalia chebula*.

**Fig. 2:**
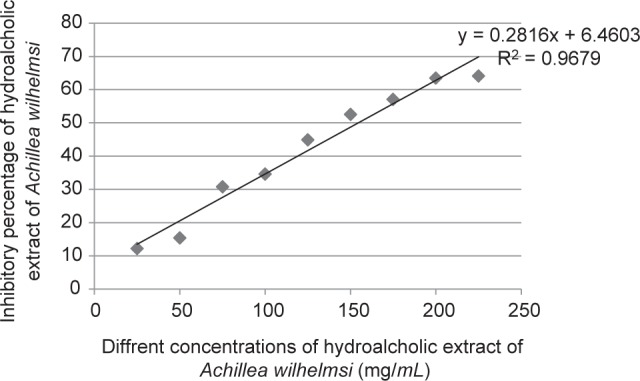
Percentage of DPPH free radicals inhibition by Achillea wilhelmsi.

The phenolic content was measured according to the standard gallic acid curve plotted by the Y = 0.1098X-2.579 formula. Accordingly, the total phenolic contents of the *T. chebula* and *A. wilhelmsii* extracts were calculated at 276.66 ± 1.45 mg GAE/g, 55.07 ± 0.295 mg GAE/g dry extract, respectively. To calculate the total flavonoid content, a standard rutin curve (formula of Y= 0.235X-8.970) was used. On this basis, the total flavonoid content of *T. chebula* and *A. wilhelmsii* extract extracts was 39.99 ± 0.192 mg and 39.14 ± 0.100 mg rutin equivalent/g dry extract, respectively. The antioxidant activities of the extracts showed that the IC50 of *T. chebula* was 4.89 ± 0.101 μg/ml (Fig. 1). and that of *A. wilhelmsii* 154.5 ± 1.01 μg/ml (Fig. 2). The antioxidant capacity of BHT as the standard material was also calculated in this study test. The IC50 of BHT was calculated 33.5 ± 0.16 μg/ml. The results showed that the antioxidant capacity of *T. chebula* was 6.85 times more than BHT and this capacity for *A. wilhelmsii* was 0.21 time more than BHT.

### 3.2 Acute toxicity results of the extracts of *T. chebula* and *A. wilhelmsii*

Acute toxicity of *T. chebula* and *A. wilhelmsii* extracts in mice with doses of 10, 156.25, 312.5 and 625 mg/kg no mortality was observed. Therefore, in the second stage, higher concentrations, i.e., 1250, 2500, and 5000 mg/kg of the extracts were used. In the animals receiving the extract at these doses, no death was also observed. Therefore, in acute toxicity phase LD50 for both extracts was over 5000 mg/kg.

### 3.3. Sub-acute toxicity results of the *T. chebula* and *A. wilhelmsii* extracts

The mortality rate of the studied animals was investigated within 14 days, which was followed up until the 30th day. At this stage, the animals received 156.25, 312.5, 625, 1250, 2500, and 5000 mg/kg doses by gavage In sub-acute phase of the study, LD50 values calculated based on the dose-response curve using Probit regression analysis exhibited that the lowest *T. chebula*’s LD50 was ≥ 2754.436 mg/kg (CI = 95%, 2438.173-3114.643). Because only one mouse died at the 5000 mg/kg of *A. wilhelmsii*, its safe dose was calculated as ≤ 5000 mg/kg.

### 3.4. Results of histopathological investigations

**Fig. 3:**
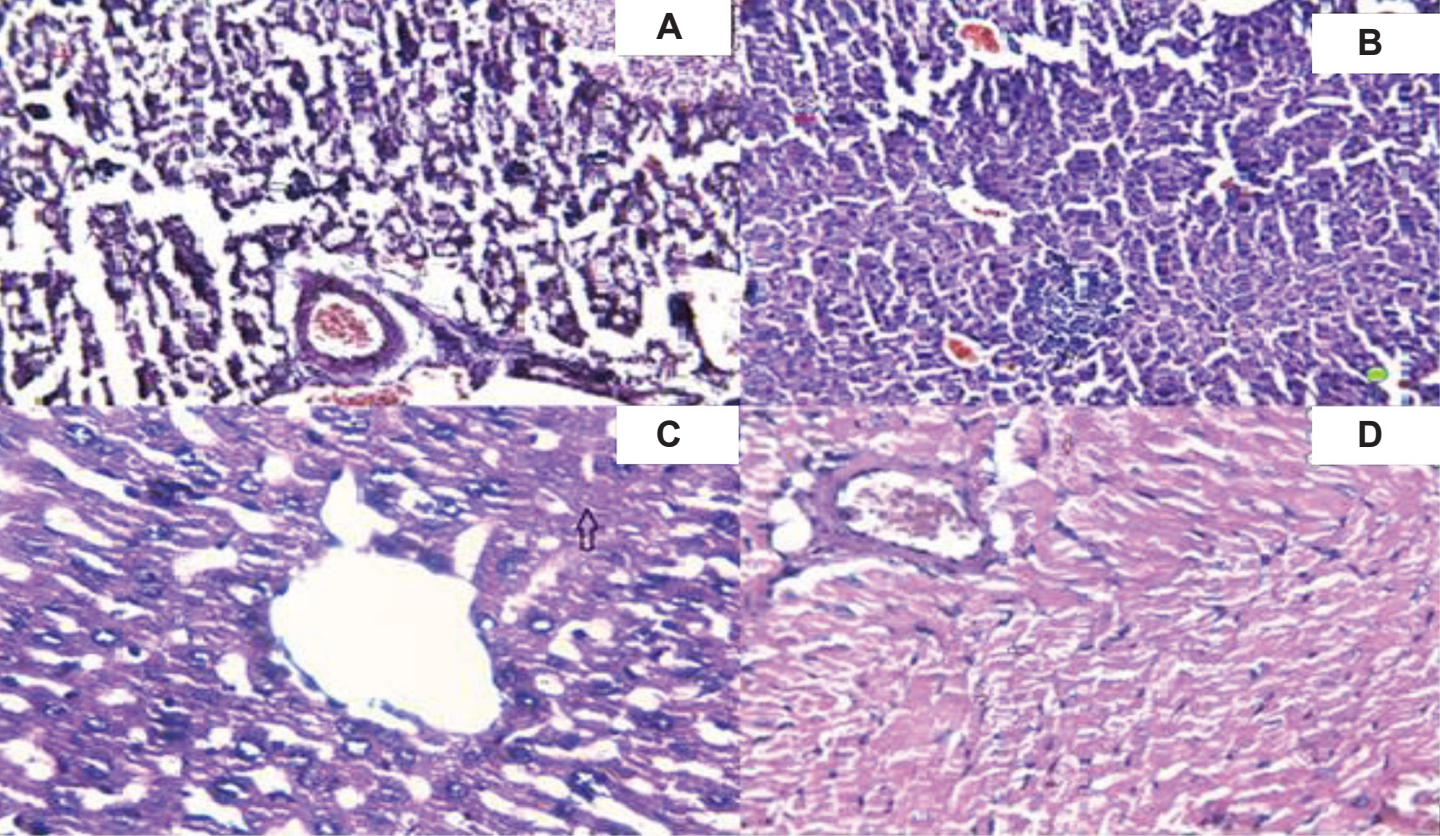
Pathologic changes in the liver (A), Kidney(B) and Heart(C) tissues in *Terminalia chebula* treated-group and the liver tissue(D) of *Achillea wilhelmsii* treated group. (H & E × 100)

Microscopic examinations showed pathological hepatic, cardiac and renal changes in the mice receiving 5000 mg/kg hydroalcoholic *T. chebula* extract. In liver tissue sections, hepatic central venous dilatation with mild hyperemia and the accumulation of acute inflammatory cells (neutrophils) were observed as focal masses that were associated with the death of liver cells (Fig. 3-A). In the kidney, atrophy and wrinkling of glomeruli (Arrow) and degeneration of renal tubules were observed (Fig. 3-B). The heart tissue sections of the subjects showed cytoplasmic deformation and striated myocytes (Arrow) along with interstitial edema (Fig. 3-C). The animals received *A. wilhelmsii* extract, at 5000 mg/kg, certain changes in the liver, including regional necrosis around the central hepatic vein (Region 3-Arrow) and the hyperemia of the central hepatic vein (Asterisk), which is exclusively vulnerable to ischemic injury were observed. (Fig. 3-D) However, no clear histopathological changes were observed in kidney and heart tissue sections of mice that received the hydroalcoholic extract of *A. wilhelmsii* [35].

## 4. Discussion

Given the growing application of medicinal plants to treat diseases and numerous pharmacological researches, the toxic properties of herbal drugs, along with their therapeutic properties need to be investigated; however, in some studies, the toxic effects of these plants have been studied. This study was therefore aimed at investigating the acute and sub-acute toxic effects of *T. chebula* and *A. wilhelmsii* extracts. In this study, the phenolic and flavonoid contents and antioxidant activity of the studied extracts were measured. According to the results, a significant correlation was observed between the antioxidant properties and total phenolic contents of the extracts of *T. chebula*, and *A. wilhelmsii* hydroalcoholic extract of *T. chebula* exhibited more potent antioxidant capacity, compared with *A. wilhelmsii*, due to its high total phenolic content (6.8 vs. 0.21 BHT). Basically, antioxidant properties increase with an increase in total phenolic content. Phenolic compounds, with high molecular weight, have a significant potential for scavenging free radicals, which depends on the number of aromatic nuclei and the nature of the hydroxyl groups influencing them. In this study, the extract of *T. chebula* was shown to be more antioxidant due to its comparatively more phenolic compounds. DPPH radicals are free, stable, organic, and nitrogenous radicals that are widely used for scavenging free radicals [36, 37]. The study of Ali Mirzaei et al. reported the anti-oxidant capacity and total phenolic content of *A. wilhelmsii* were low, and the study of Tupe et al. confirmed the high amount of total phenolic compounds and the high antioxidant capacity of *T. chebula* [38, 39]. In this study, the minimum lethal doses (LD50 values) of hydroalcoholic extracts of *T. chebula* and *A. wilhelmsii* in BALB/c mice were determined in the acute phase, no mortality occurred among the animal subjects. However, in the sub-acute toxicity phase, mortality was observed in mice receiving 2500 and 5000 mg/kg of *T. chebula* extract. In this regard, the minimum lethal dose of hydroalcoholic extracts of *T. chebula* was determined to be 2754.43 mg/kg. However, in the mice receiving the *A. wilhelmsii* extract, only one case of death was seen at 5000 mg/kg. Therefore, the minimum lethal dose of *A. wilhelmsii* extract was ≤ 5000 mg/kg. addition, the histopathological examination of various organs in this phase of the study showed that in mice receiving 5000 mg/kg of hydroalcoholic *T. chebula* extract, certain lesions developed in the liver tissue, which can be due to cell responses to the cytotoxicity due to the extract [35]. Our study showed that administration of the highest dose of *T. chebula* extract caused pathological changes in kidney, liver, and heart tissues, which can be due to the existence of high level of phenolic content in the extract. Studies have shown that phenolic compounds at high concentrations can exhibit peroxidase-like behaviors and cause damage [40]. However, there were no significant pathological changes in kidney and heart tissues of mice treated with hydroalcoholic extract of *A. wilhelmsii*, and only regional necrosis around the central hepatic vein in the liver tissue was observed.

## 5. Conclusion

In acute toxicity phase, the administration of 5000 mg/kg of hydroalcoholic *T. chebula* and hydroalcoholic *A. wilhelmsii* extracts is safe. Regarding sub-acute toxicity, *T. chebula* extract at 2754.436 mg/kg and *A. wilhelmsii* extract at a dose of at most 5000 mg/kg is safe. Therefore, in future studies, safe doses can be selected by investigating the minimum lethal doses (LD50 values) of hydroalcoholic extracts of *A. wilhelmsii* and *T. chebula*.

## Conflicts of interest statement

The authors wish to disclose no conflicts of interest.
